# A new surgical ventricular restoration technique to reset residual myocardium's fiber orientation: the "KISS" procedure

**DOI:** 10.1186/1750-1164-3-6

**Published:** 2009-06-23

**Authors:** Marco Cirillo

**Affiliations:** 1 Cardiovascular Department, Heart Surgery Unit, Poliambulanza Foundation Hospital, Brescia, Italy

## Abstract

**Background:**

The history of surgical reconstruction of the left ventricle after an anterior myocardial infarction shows an evolution of techniques which tend to a more and more physiologic restoration of ventricular shape and volume, with increasing attention to the orientation of myocardial fibers.

**Methods:**

We set a new surgical procedure for endoventricular patch reconstruction technique with the aim to rebuild a physiologic shape and volume of the left ventricle caring about realignment of myocardial fibers orientation. Peculiarities of this reconstruction are the shape of the patch (reduction of minor axis compared with currently used oval-shaped patch) and the asymmetrical way of suturing it inside the ventricle.

**Results:**

We present a detailed description of operative steps of this procedure, and we add some relevant surgical hints to clarify its peculiarities. Most of the patients operated on with this technique showed the original renewal of apical rotation and left ventricular torsion as specific index of the restoration of physiologic fiber orientation: we report an exemplary case of at-sight recovery of apical rotation in the operating room.

**Conclusion:**

This technique can represent a reproducible new way to realign myocardial fibers in a near-normal setting, improving the physiological restoration of ischemically injured left ventricle. It could be also the basis to reconsider surgical treatment for heart failure.

## Background

Surgery for left ventricular anterior reconstruction in the setting of ischemic cardiomyopathy has a long history. It has evolved over the years from linear closure of large aneurysms to present geometric reconstruction by means of endoventricular patch [1-4]. Surgical and clinical outcome have extensively been revised in many retrospective studies, highlighting a variability in the results of this surgical treatment [5-10]. The internal border zone between normal and infarcted tissue was usually encircled by the Fontan purse-string, a running circular suture with the aim to approach residual walls and reduce the tissue gap to be closed by the patch. The original circular patch used in the reconstruction of the residual tissue gap has been replaced by an oval-shaped patch [11,12], able to assure a better ventricular shape and volume, which have been pointed out as two of the major determinants of steady long-term results [13-16]. Thanks to the improving outcomes of this approach, a new chapter of left ventricular volume reduction has opened [17-19], supported by new and accurate studies on left ventricular geometry and function [20], with particular interest in myocardial fiber disposition [21,22].

## Methods

We here describe a detailed surgical plan of a novel technique aiming at realigning residual myocardial fibers in a physiologic setting. It is a logical consequence of the state of the art of this surgical treatment and derives from studies about the pathological changes that intervene in the myocardium after an acute infarction. The technique has been improved since 2002, until its standard application in 21 patients (2005–2008). As an indirect evidence of restored fiber disposition, we observed in several patients, immediately after the surgical correction in the operating room and during the follow-up, the renewal of apical rotation and, consequently, of left ventricular torsion, a peculiar movement of the normal heart that optimizes ejection efficiency and energy expenditures: it was lost due to the dilation of the apex and to the loss of contractile myocardium and its renewal, although postulated, was never demonstrated before in any technique of ventricular reconstruction [23,24].

Failure to obtain its renewal during a so long clinical history of endoventricular reconstruction testifies that it does not exclusively depend on revascularization and reshaping itself, but on specific solutions addressing fiber rearrangement.

## Surgical procedure

We called this surgical procedure "KISS", acrostic for "**K**eep f**I**bers orientation with **S**trip patch re**S**haping": the name underlines its peculiar action to let normal walls "kiss" together, restoring anatomic fibers' contiguity and orientation. Peculiarities of this reconstruction are:

1. the shape of the patch (reduction of minor axis compared with currently used oval-shaped patch, tailored major axis and arrow-shaped ends);

2. the absence of Fontan purse-string;

3. the asymmetrical way of suturing it inside the ventricle.

Preliminary phases are common to the standard operative management: median sternotomy, mild hypothermic (33°C) cardiopulmonary bypass, aortic cross clamping, intermittent cold blood cardioplegia and warm reperfusate. Left ventricle is opened, as usually, through an anterolateral incision two cm aside the left anterior descending artery.

Endoventricular reconstruction surgery was performed by means of a very narrow, strip-shaped patch, with arrow-shaped ends, whose minor axis is less than one cm wide and major axis is proportional to the width of infarcted area. The patch used was made either by glutaraldehyde-treated autologous pericardium or by knitted Dacron (Hemashield Platinum Finesse, 0.8 × 7.6 cm, Boston Scientific, Natick, MA, USA).

### Operative steps

**Figure 1:**
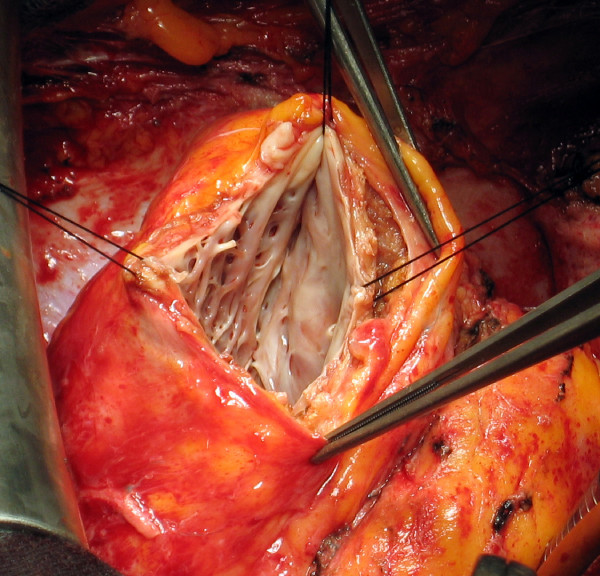
**Apex Stitch**. Angle between the end of necrosis and the septum: it corresponds to the lower end of the patch and will be the new apex (apex stitch).

**Figure 2:**
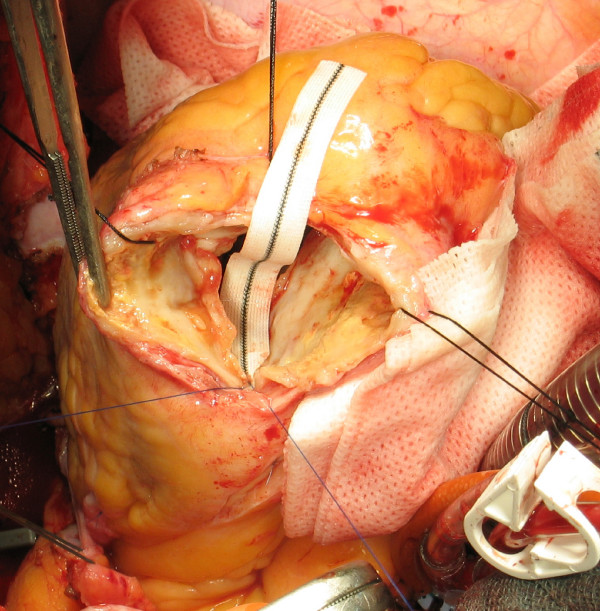
**High Stitch**. Upper limit of fibrotic tissue in the interventricular septum: it is the upper end of the patch (high stitch).

**Figure 3:**
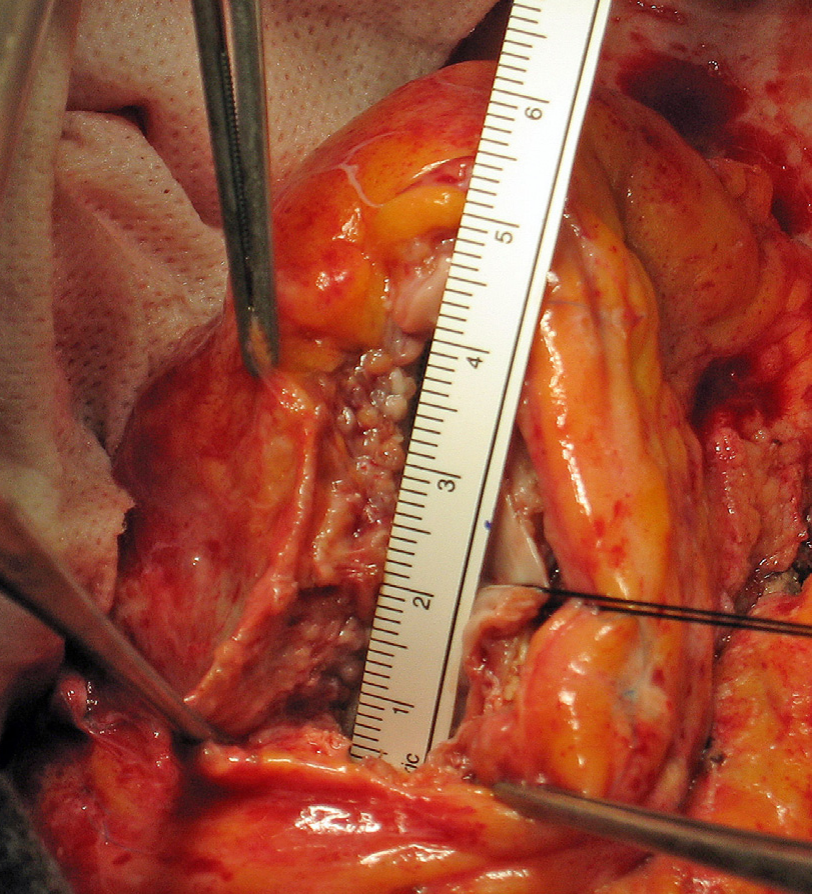
**Patch measure and tailoring**. The distance between these two points (the high and the apex stitches) is the length (major axis) of the patch that is then tailored according to this measure with an arrow-shaped end.

**Figure 4:**
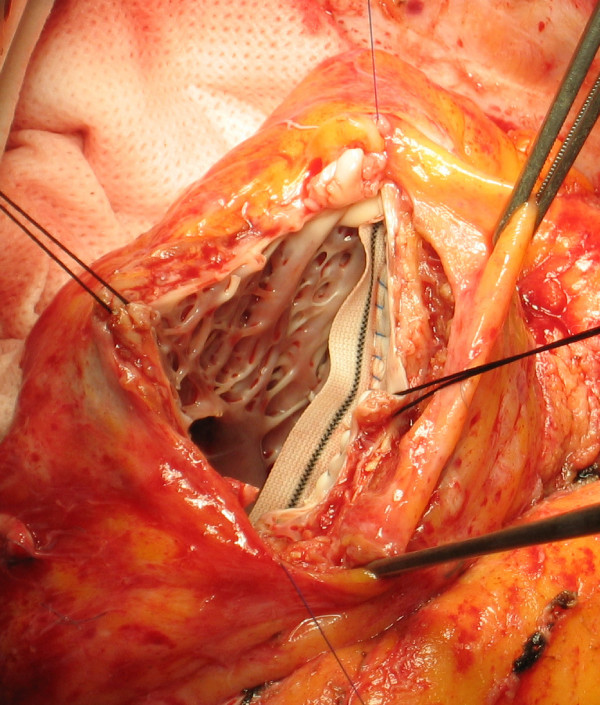
**Septal Suture**. All the inferior (septal) rim of the patch is sutured along the basal border of the fibrotic interventricular septum, from the high to the apex stitch (patch-tissue equivalence).

**Figure 5:**
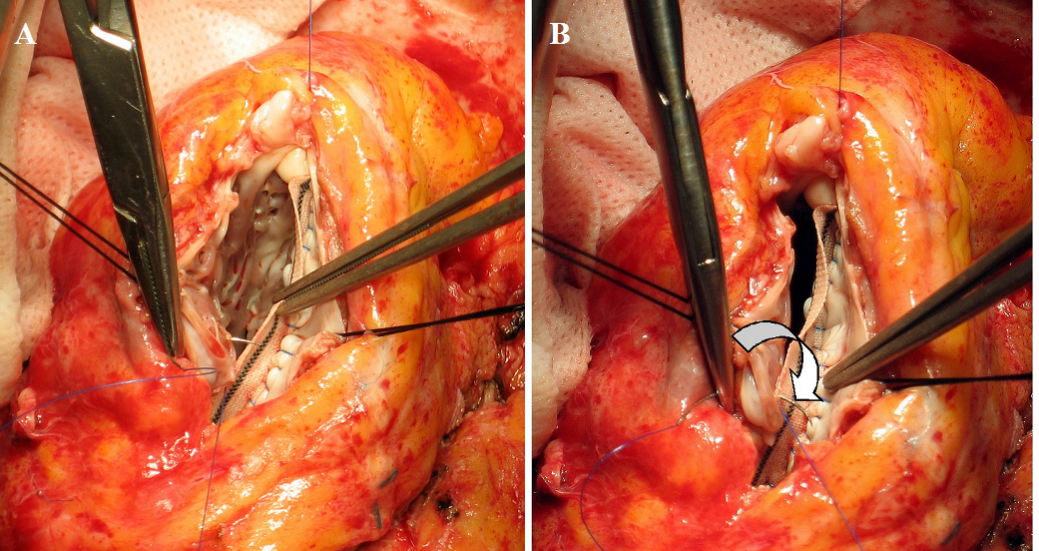
**Lateral Suture**. The lateral border zone between fibrotic and normal tissue of the ventricular wall is secured to the superior (lateral) rim of the patch (patch-tissue mismatch). Panels A and B depict the stretching of the suture that approaches and fits the lateral wall to the patch. The arrow in panel B shows how the suture redirects fiber orientation, approaching a farer point of the myocardial wall to a nearer point on the patch.

**Figure 6:**
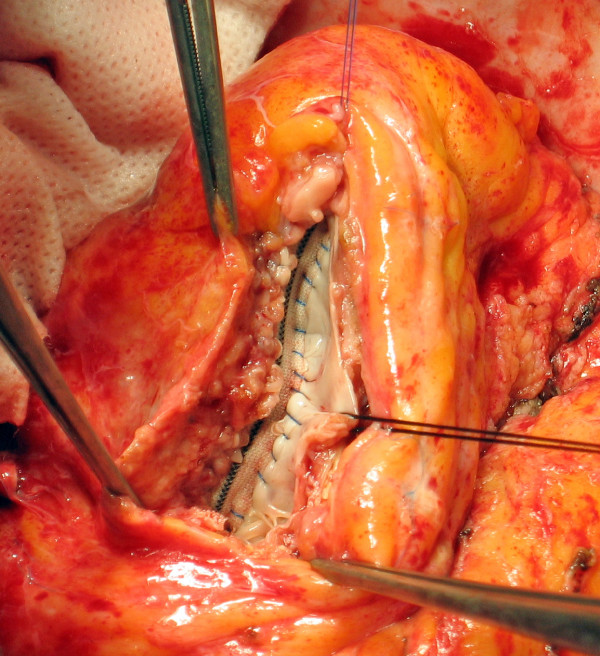
**Final appearance**. The two sutures end at the Apex stitch and are tied together.

**Figure 7:**
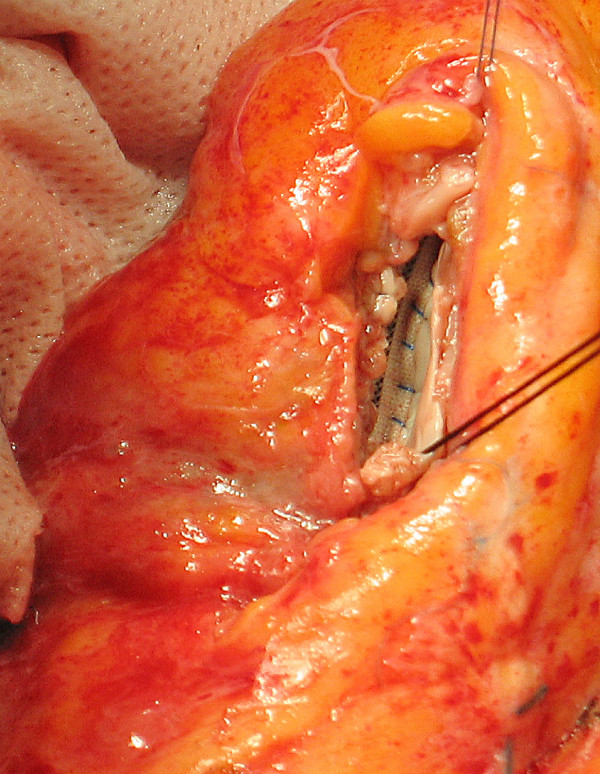
**Ventriculotomy closure**. Ventriculotomy was closed by overlapping the free edges; note the "goffered" lateral wall, due to length compensation obtained by the suture.

1. Once opened the left ventricle, boundaries between normal and fibrotic tissue, if present, are identified; in case of akinetic, non-fibrotic area, thinning of the area and information derived from echocardiographical and/or magnetic resonance studies are used to identify the limits from normal myocardium.

2. The Fontan purse-string is not used, to avoid a circular disposition of the border zone between normal and fibrotic muscle that alters the spatial geometry of myocardial walls and forces myocardial fibers to a non-physiologic orientation; in this way, we let the suture to redirect fibers, without any pre-conditioned deformation.

3. The limit of the fibrosis near the apex is then identified: this is the distal end of the patch and will form the new apex; it corresponds to the angle between the end of necrosis and the septum and is defined as "Apex stitch" (Figure 1).

4. The limit of fibrotic tissue in the interventricular septum (near the aortic valve) is identified: this is the upper end of the patch and is defined as "High stitch" (Figure 2).

5. The distance between these two points (the high and the apex stitches) along the interventricular septum is the length (major axis) of the patch that is then tailored according to this measure with arrow-shaped ends (Figure 3).

6. The upper end of the patch is secured to the High stitch by a 4.0 polypropylene monofilament (Prolene, Ethicon), tied at its half length with few knots (Figure 2).

7. All the septal rim of the patch is then sutured along the basal border of the fibrotic interventricular septum, from the high to the apex stitch, with a continuous running suture using the 4.0 polypropylene monofilament: in this suture there is almost a patch-tissue equivalence, that is the length of the patch and the length of the myocardial wall are quite equal (Figure 4).

8. The lateral rim of the patch is then secured to the lateral border zone between fibrotic and normal tissue of the ventricular wall with the other half of 4.0 monofilament: this line is widened by the dilation and the curvature of the left ventricle, so the length of the patch is smaller than the length of the ventricular wall (patch-tissue mismatch) (Figure 5A, B): surgeon must fit the length of lateral wall to the length of the patch, shrinking in this way the ventricular wall to a shorter line, that is, forcing the orientation of residual myocardium (and myofibers' lamina) towards a tighter disposition, compensating the dilation and spacing due to akinetic/dyskinetic portion of the wall.

9. The two sutures end at the Apex stitch and are tied together (Figure 6).

10. Ventriculotomy was closed by overlapping the free edges in a vest-over-pants fashion, in order to occlude the excluded chamber, with or without a felt strip to reinforce the suture (Figure 7).

### Relevant surgical hints

• The first running suture ("septal") has quite the same length as the patch: this part of the ventricular wall usually becomes thinner rather than dilated, due to the anatomy of the septum, embedded between the two ventricles, with a dual layers structure vascularized by two coronary systems; in some cases is possible to compensate a greater dilation of this part with the suture, stretching the residual myocardium to a shorter patch, but in most of the cases there is a patch-tissue equivalence.

• Position of the new apex is often determined by the end of the necrotic tissue; otherwise, its relocation should be guided by normal geometry; normal values of diastolic left ventricular length are 80 ± 9 mm [25], so this should be the maximum value of the new chamber, adding patch length to the length of the normal upper part of interventricular septum (from aortic valve to akinetic/fibrotic septum); new apex should also be near the level of right ventricular apex, as is in the normal heart.

• Given that usually we can reach the upper septum at 2–3 cm from the aortic valve, it follows that the length of the patch should be 5–6 cm in most cases; the end of right ventricle is another reference point to set the new apex.

• The second suture ("lateral") forces the lateral wall to fit to the patch, stretching myocardial fibers towards a shorter line, thus realigning them in a narrower setting, representing the key characteristic of this technique. For this reason the lateral sutured myocardial border appears "goffered" at the end of the procedure, to compensate the discrepancy between patch and myocardial suture rims (Figure 7).

• Converting the setting to a geometrical figure, as previously described [26], we have a circular segment in which the chord is the basal line of the fibrotic interventricular septum and the arc is the semicircular line of lateral border zone between fibrotic and normal tissue of the ventricular wall; the surgical suture approaches the arc to the chord, reducing the arc's length of about one half.

• The gross realignment of residual myocardial fibers at the border zone in order to have a recovery in their oblique spatial orientation can only be obtained by a narrow (small minor axis) patch, which forces the realignment, while a wider (great minor axis) patch tends to maintain fibers direction in the setting of a dilated left ventricle, eliminating the length compensation induced by this technique.

• The use of a patch is of primary importance for fibers' realignment: despite this technique could seem like a linear suture, only the use of the patch makes to grade the stretching of the two sutures possible, differentiating the fitting of the two rims (septal and lateral) to the relative border of the patch; otherwise, it would be impossible to adjust different fibers' orientation along inferior septum (less dilated = fibers less divergent) and lateral wall (more dilated = fibers more divergent).

• The key features of this surgical technique are the small short axis of the patch and the asymmetrical suture at the two sides of the patch: the first one approaches residual myocardium as close as possible; the second differentiates the reconstruction of the dilated border zones to re-establish a physiologic contiguity of myocardial lamina.

## Results

### Overall Results

Twenty-one patients were operated on with this technique. Mean age was 60.8 ± 12.5 years. Eleven of them were male. Eight patients had a mitral regurgitation ≥ grade 3 and were contemporarily treated with mitral ring anuloplasty. All patients received complete coronary revascularization. Patch dimensions were 1.0 ± 0.3 × 5.9 ± 1.0 (short × long axis, cm). No hospital deaths were reported. At a mean follow-up time of 1.95 ± 0.96 years, all patients were alive and in functional NYHA class = 1.3 ± 0.5. Two-dimensional speckle tracking imaging, a reliable ecocardiographical tool for rotation movements of the heart, was used to assess left ventricular torsion in presence of a good acoustic window (16 out of 21 patients). Echocardiographical data are reported in Table 1.

**Table 1:** Overall series data

	Preop	Follow-up data (1.95 ± 0.96 years)
EDV	222.8 ± 66.7	136.2 ± 39.3

EDVI	122.0 ± 34.4	73.1 ± 35.2

ESV	159.3 ± 50.7	88.0 ± 31.0

ESVI	87.5 ± 27.9	35.2 ± 16.1

EF	27.9 ± 6.9	36.4 ± 8.5

LV TORSION	2.5 ± 4.6	7.7 ± 3.4

EDV = end-diastolic volume (ml); EDVI = end-diastolic volume index (ml/m^2^); ESV = end-systolic volume (ml); ESVI = end-systolic volume index (ml/m^2^); EF = ejection fraction. LV torsion is reported in degrees.

### Exemplary case

A 52-years-old male patient, ZD, had the acute episode of myocardial infarction on August 2001, treated with thrombolysis. On September 2001 he performed a coronary angiography showing critical stenoses of left anterior descending (LAD) and right coronary arteries. He then underwent off-pump surgical revascularization by means of three coronary grafts (diagonal, anterior descending and right coronary arteries). The left ventricular function was already compromised (EF = 30%). Postoperative course was uneventful. Since 2004 he suffered from several episodes of heart failure. On July 2004, coronary re-angiography showed a mild stenosis of the graft on LAD (50%) and left ventricular dysfunction (EF = 28%) with anterolateral aneurysm. Grade 4 mitral regurgitation was evident. Ventriculography showed a completely upset LV geometry with consequent loss of ventricular apex and torsion movement (Additional file 1). On May 2005, the patient was reoperated (remodeling time from acute infarction to surgery = 49.7 months). Endoventricular reshaping was performed as described. Patch dimensions were 1 × 6 cm. A 30-mm rigid ring mitral anuloplasty was associated. Left anterior descending artery was grafted by a saphenous vein. Once weaned from extracorporeal circulation, left ventricle showed the renewal of apical rotation and, as a consequence, of left ventricular torsion, as evident in Additional file 2. Echocardiographical data are reported in Table 2. Patient was discharged to a rehabilitation hospital eight days after the operation and is now in first functional NYHA class. A 3D full volume echocardiography was performed at late follow-up (19 months), showing a still present apical rotation at several apical slices (Additional file 3).

**Table 2:** Exemplary case data

	Preop	Postop 7 days	Postop 19 months
EDD	75	65	68

ESD	59	54	51

APICAL DIAMETER	70	36	35

LLD	82	79	78

LLS	82	76	76

EDV	256	160	136

EDVI	135	84	69

ESV	150	120	95

ESVI	79	62	48

EF	28	35	38

WMSI	2.88	1,88	1,56

MR	4	0	0

NYHA	4	1	1

EDD = end-diastolic diameter (mm); ESD = end-systolic diameter (mm); APICAL DIAMETER = internal diastolic diameter two centimeters above the ventricular apex (mm); LLD = diastolic longitudinal length (mm); LLS = systolic longitudinal length (mm); EDV = end-diastolic volume (ml); EDVI = end-diastolic volume index (ml/m2); ESV = end-systolic volume (ml); ESVI = end-systolic volume index (ml/m2); EF = ejection fraction; WMSI = wall motion score index; MR = mitral regurgitation; NYHA = New York Health Association class.

Due to a poor echocardiographical window, this patient was not studied by 2D speckle tracking echocardiography. Some of patients studied by this method were reported as single clinical cases [27,28], demonstrating the renewal and the persistence during time of apical rotation and LV torsion.

## Discussion

### Left ventricular structure and alterations induced by myocardial infarction

The left ventricle is characterized by a unique myocardial tissue structure [29,30]. The necrosis induced by myocardial infarction alters the myocardial continuum, forcing the whole ventricle to work at a lower efficiency level. Necrotic area spaces out the residual normal tissue and negative remodeling following the acute episode [31] completely alters ventricular geometry and mechanic. Nevertheless, it is demonstrated from *in vivo *studies that myofibers orientation in the thickness of normal myocardium is not changed after myocardial infarction [32] and that transmural courses of fiber orientation angles near infarct zones were similar to those of normal myocardium [33]. This means that infarct site demarcates at the end of necrotic process, with an extension related to the tributary area of the occluded coronary vessel and to the wavefront phenomenon of ischemic cell death [34]: definitive boundaries are characterized by a still normal myocardium interlaced to the necrotic region. This is shown also in gross histological samples [35]: a normally structured myocardium ends without graduation where starts the fibrotic infarcted tissue. It is also evident that alterations occurring in myocardium adjacent to an infarction consists with myocytes elongation and myofiber rearrangement (slippage), as well as changes in orientation of the laminar structure of the ventricular wall [36].

The key point to understand how myocardial structure is altered by coronary vessel occlusion is the relative position of epicardial coronary vessel and fiber orientation.

**Figure 8:**
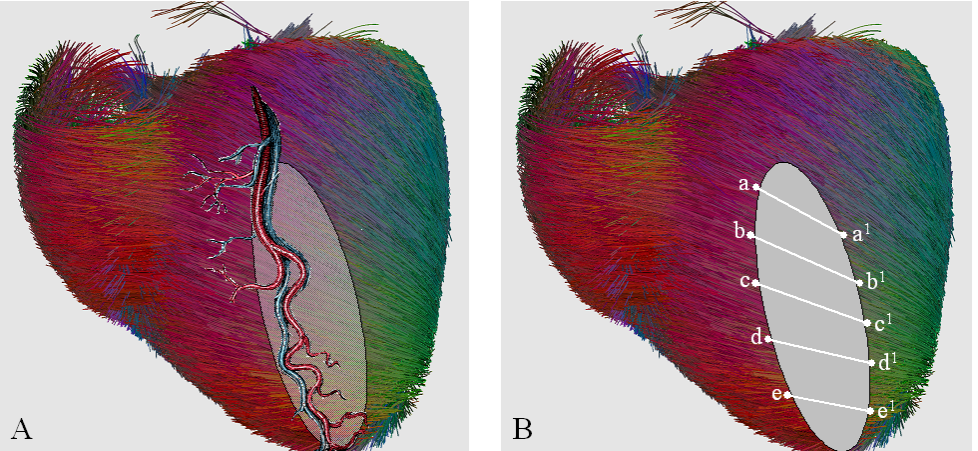
**Coronary vessels and fiber orientation**. A: Epicardial course of coronary arteries crossing fiber orientation. The shaded area depicts transmural necrosis induced by coronary vessel occlusion which concerns a sector of myocardium made by several strata of differently oriented myocardial fibers and ends where the territory of another coronary vessel begins. B: At the border zone, still normal myocardium with still normal fiber orientation interlaces with the necrotic tissue that lost its 3D fiber structure. Necrotic tissue discontinues contiguity of myocardial fibers, although they remain normally oriented in the thickness of normal myocardium (correspondence of points "a" and "a1", "b" and "b1" etc). Fiber picture obtained by the DTI Track module of MedINRIA software [45].

Coronary arteries have an epicardial course not to be squeezed during systole, while fiber orientation is changing throughout myocardium thickness, from epicardium to endocardium, following a continuous angular gradient which is crossed more or less perpendicularly by vessels direction (Figure 8A). The transmural necrosis induced by coronary vessel occlusion concerns a sector of myocardium made by several strata of differently oriented myocardial fibers and ends where the territory of another coronary vessel begins (Figure 8A, shaded area). At the border zone, still normal myocardium with still normal fiber orientation interlaces with the necrotic tissue that lost its 3D fiber structure. Necrotic tissue discontinues continuity and contiguity of myocardial fibers, although they remain normally oriented in the thickness of normal myocardium (Figure 8B, correspondence of points "a" and "a^1^", "b" and "b^1^" etc).

The infarcted area alters regional and then global function of the left ventricle, expands and dilates the ventricular chamber and dislocates adjacent, still anatomically normal, non-infarcted myocardium. Consequently, fibers in the normal myocardium are displaced by the necrotic, thin, akinetic/dyskinetic portion of ventricular wall and (although normally oriented inside the residual wall) their orientation and spatial disposition is completely upset.

On the other hand, it has been demonstrated that surgical intervention could effectively alter (positively or negatively) the helix angles in regions adjacent to repair [32].

### KISS reconstruction

Surgical restoration replaces the infarct scar, and tries to modify ventricular geometry in such a way as to recreate a nearly normal ventricle. The described procedure reduces the distance from the contractile myocardium thanks to the narrow patch that absorbs the stress of the suture, acts as a support for the suture lines and guides the restoration of myocardial wall contiguity and fibers' orientation. A narrow patch also helps to rebuild near-normal ventricular volumes because it does not add any volume itself. Comparing the left ventricle to a prolate ellipsoid, small variations in the minor axis correspond to great changes in ventricular volume (1 cm less in the minor axis produces a reduction in volume of about 30 ml). In this way, major determinants of normal physiology are restored: elliptical shape, near-normal volume, small residual akinesia, contiguity of contractile myocardium, spatial geometry and fibers orientation. Complete revascularization and, when required, the competence of mitral valve crown the physiologic restoration.

Aiming the ventricular restoration at the realignment of residual fibers could overcome the endless contest about akinetic and dyskinetic ventricular walls. We should assume that akinesia and dyskinesia are different stages/states of the same disease and both result in a non-contractile zone of the myocardium. From a strictly functional point of view, both interrupt myocardial continuum, lead to fibers disarrangement and ventricular remodeling, so both are responsible of the same pathophysiology. We should then reconsider that the border zone of myocardial infarction is the limit between fibers that lost and fibers that preserved their anatomical and physiological arrangement. This is the consequence of the ischemic disease, and in case of akinetic areas, in which the border zone is not marked by fibrotic degeneration, it is visually less evident, but functionally has the same effect.

Finally, also a non-trasmural infarction can damage myocardial continuum in such a way to alter its functionality, though zones of normal fibers remain in the thickness of the infarcted region. These residual fiber islands, embedded in the fibrotic tissue or simply adjacent to it, can not work properly because they are blocked by the non-contractile cells, and the physiologic fiber interlacement that produces the contraction is lost: they do not act on normal adjacent fibers, propagating the contraction, but their action extinguishes against non-contractile tissue. In these cases, function of myocardial wall is the result of the delicate balance between contractile and non-contractile fibers.

### Left Ventricular Torsion

Since 1911 [37] left ventricular torsion was recognized to be essential for ventricular ejection and many studies confirm its importance as an index of normal ventricular function in physiologic and pathologic settings [38-41]. The twisting motion of the left ventricle about its long axis results from the contraction of the opposite, obliquely oriented epicardial and endocardial fibers that produce the contemporary clockwise rotation of the base and counterclockwise rotation of the apex, obtaining the torsion of the heart along its long axis. Loss of myocardial structure in anterolateral region, chamber dilation, volume overload and loss of the ventricular apex, lead to the loss of apical rotation and, consequently, of left ventricular torsion.

The evidence of the recovery of left ventricular torsion is an indirect but striking demonstration that fibers should be reset to a physiologic orientation with the described endoventricular reshaping. The evidence of torsion recovery corresponds to a more physiologic rearrangement of myocardial fibers. We observed the renewal of ventricular torsion ever since the end of the surgical procedure in the operating room, immediately after the surgical correction: so it must be due to a mechanic correction of fibers orientation and not only to a progressive positive remodeling after surgery.

### Current Limitations and Future Developments

At present, we have only indirect, "gross" evidence of fiber realignment, but we can not correct fiber disposition in order to obtain a "histological" realignment. Fast improvement in medical imaging techniques able to visualize fiber orientation (diffusion tensor magnetic resonance, limited until now by heart movement) will be very useful in this topic and will probably lead to a preoperative assessment full of key data for the surgeon. Some advanced technology laboratories [42,43] are already studying the use of integrated imaging techniques for accurate planning of surgical procedures (for example, tumor removal in the brain with 3D visualization of cerebral fibers, tissue and structures). It would be reasonable to foresee a 3D surgical planning of ventricular reshaping with reference points for fiber disposition that will guide the surgeon during the reconstruction. A so called "fiber-based" therapy could open unexpected improvements in the surgical treatment of the failing heart. For example, the Batista operation and the surgical reshaping in the setting of dilated cardiomyopathy had not the expected diffusion, perhaps because volume reduction did not take care of fiber alignment at the suture site, but caused itself a fiber disarrangement, suturing together non-contiguous parts of ventricular walls with different fiber disposition. A fiber-based physiological view could also help us in the evaluation and/or optimization of medical therapies, mainly in case of new drugs or of non-responder patients.

Finally, basic science implications of fiber-based left ventricular function could let us use an old knowledge that was forgotten until now, due to the imaging limitations and to the complex 3D anatomy of myocardium, but that could represent the basis for a new approach to the failing heart.

## Conclusion

The knowledge of anatomical structure of left ventricle is very old [44] but it has never been applied in clinical practice. The described endoventricular reconstruction tends to grossly realign fibers in a more physiologic manner, approaching them together and re-establishing their oblique disposition and orientation, compensating in this way the spacing and the anomalies induced by myocardial infarction. The evidence of the renewal of left ventricular torsion could open the way to implement the evaluation of the results of this surgical procedure. Variability in outcome could be overcome with a multifactorial approach to restoration taking care of all aspects of normal physiology of ventricular function, in which fibers-based procedures will have a key role. Future development in medical imaging will probably confirm and support the main role of fibers' disposition in the surgical treatment of failing hearts.

## Competing interests

The author declares that they have no competing interests.

## Authors' contributions

MC set the theoretical bases and conceived the surgical procedure, performing all of the operations. The described surgical technique and its peculiar result (the original, never described before renewal of left ventricular apical rotation and torsion) are to be considered his own intellectual property.

## Supplementary Material

Additional file 1 **Exemplary Patient: preoperative left ventriculography**. Preoperative left ventriculography of the exemplary patient with large dyskinesia: normal geometry and torsion movement are completely lost. Click here for file

Additional file 2 **Exemplary Patient: renewal of left ventricular torsion at the end of operation**. Head view of the heart at the end of reconstruction surgery of the same exemplary patient, normal and slow motion of the same sequence. Cardiopulmonary bypass is off. Ventilation is temporarily off. The counterclockwise torsion movement towards the right ventricle is evident, marked by the displacement of the saphenous graft on left anterior descending artery. Click here for file

Additional file 3 **Exemplary Patient: late full volume echocardiography**. Simultaneous display of nine short axis views generated from an apical full volume acquisition with 4D mode (Vivid 7, GE Medical Systems, Norway) of the exemplary patient 19 months after the operation. Apical rotation is evident towards the right ventricle. Click here for file
